# The health-promoting lifestyle behaviors of healthcare employees in Qatar – A cross-sectional comparative study

**DOI:** 10.5339/qmj.2024.74

**Published:** 2024-12-27

**Authors:** Shamja Sofia Razzakh, Rajvir Singh, Bilal Uddin Khan, Nesiya Hassan

**Affiliations:** ^1^Department of Medicine, Hamad General Hospital, Hamad Medical Corporation, Doha, Qatar; ^2^Academic Health System, Hamad Medical Corporation, Doha, Qatar; ^3^Nursing and Midwifery Research Department, Hamad Medical Corporation, Doha, Qatar *Correspondence: Shamja Sofia Razzakh. Email: srazzakh1@hamad.qa

**Keywords:** Health promotion, health education, health-promoting lifestyle behaviors, risk group, healthcare employees, Qatar

## Abstract

**Background:** The World Health Organization (WHO) considers health to be a fundamental human right. Health is a resource that enables people to lead productive lives on an individual, social, and economic level. In Qatar, there are a limited number of studies addressing the health-promoting lifestyle behaviors of healthcare employees.

**Aim:** The aim of this study was to compare the health-promoting lifestyle of at-risk and non-risk groups of employees working in Hamad Medical Corporation (HMC), the largest secondary and tertiary healthcare provider in the State of Qatar.

**Methods:** This was a cross-sectional comparative research study of all categories of healthcare employees working in HMC facilities. Participants with a body mass index (BMI) >30 kg/m^2^, smokers, or those with pre-existing non-communicable diseases (NCDs) were classified as the at-risk group, and individuals without any of these factors were classified as the non-risk group. Data were collected through an online survey using an adopted scale, Adolescent Health Promotion Short Form (AHP-SF), after approval by the Institutional Review Board (IRB) of HMC.

**Results:** The age of the participants ranged from 22 to 69 years and the majority of them were female (64.07%). Most of the respondents were overweight or obese, accounting for 42.99% and 26.68% of the sample, respectively. Interestingly, 87.64% of the participants were non-smokers and approximately 70% had no chronic diseases. The overall AHP-SF score was 60.01/84 ± 12.32, with the highest score from the “life appreciation” subscale (12.68/16 ± 2.84) and the lowest score from the “exercise” subdomain (7.05/12 ± 2.93). Five subdomains – nutrition, social support, health responsibility, exercise, and stress management – of the AHP-SF scale showed no significant statistical differences between at-risk and non-risk groups. However, the “life appreciation” scale showed significant statistical differences between the at-risk (12.91/16 ± 2.69, *p* = 0.04) and non-risk (12.42/16 ± 2.98) groups. The AHP-SF scores varied significantly across the participants’ regions of origin, with Americans having the highest score (63.93/84 ± 10.67, *p* = 0.03) compared to other regions.

**Conclusions:** Healthcare employees moderately practice health-promoting lifestyle behaviors. The lowest scores were in the exercise subdomain, suggesting that more interventions are required to improve these behaviors. Healthcare organizations are ideal settings to implement comprehensive workplace wellness programs and awareness campaigns that can motivate employees to take greater responsibility for their own health and influence the wider community to adopt health-promoting lifestyle behaviors.

## INTRODUCTION

The World Health Organization (WHO) 2022 Progress Monitor Report on Non-Communicable Diseases reveals that non-communicable diseases (NCDs), such as cardiovascular diseases, cancer, diabetes, and chronic respiratory diseases, are the leading causes of death worldwide, which account for 74% of deaths worldwide.^1^ These NCDs are associated with modifiable behavioral risk factors such as tobacco use, unhealthy diet, lack of physical activity, and harmful alcohol consumption. NCDs remain a major public health challenge globally, including in low- and middle-income countries, where over three-quarters of NCD-related deaths occur. This report highlights that NCDs account for 77% of deaths in Qatar.^1^ According to the public health report, the major NCDs of public health concern in Qatar are cardiovascular diseases such as ischemic heart disease and stroke, diabetes, cancer, and chronic respiratory diseases such as asthma and chronic obstructive pulmonary diseases.^2^

The strategy for managing NCDs focuses on addressing risk factors at individual, societal, national, and global levels. This approach includes resource allocation, multi-sectorial partnerships, knowledge and information management, and innovations. Lifestyle management at the individual level is the most critical aspect of this prevention strategy. Emphasizing actions such as innovative approaches helps raise awareness about risk factor management in society, facilitate health policy decisions at the national level, and foster the development of global health strategies. The most effective prevention strategy entails lifestyle changes related to diet, physical activity, smoking cessation, and control of metabolic disorders.^3.^

A healthy lifestyle refers to the behavioral patterns that individuals adopt to maintain and enhance their health based on motivations, norms, abilities, and knowledge about healthy, stress-relieving behaviors.^4,5^ According to Wang and Geng, health is closely linked to people's lifestyles in various social contexts^5^ Furthermore, physical health and psychological well-being are closely related, and psychological well-being acts as a protective factor for health by reducing the risk of chronic diseases and promoting longevity.^6^ A healthy lifestyle and living depend on personal behavioral choices.

A comparison by professional occupation showed that healthcare workers generally follow healthier nutrition, engage in physical activity, and have greater health responsibility than individuals working in other domains.^7^ However, this trend was not observed in spiritual growth, interpersonal relationships, and stress management.

Health-promoting behaviors include maintaining a healthy diet, regular exercise, stress management, and fostering positive interpersonal relationships. A health-promoting lifestyle strongly predicts an individual's overall health status.^8^ Therefore, health promotion strategies at the individual, community, and public levels are necessary to support people in adopting healthier lifestyles.^9^ Health promotion activities are interrelated with many factors, such as gender,^10^ current health status,^11^ educational status, economic level,^12^ chronic diseases, duration of illness,^13^ language barriers, and cultural beliefs.^14^

The Ministry of Public Health (MoPH) Qatar implemented a program as part of the National Health Strategy 2018–2022 “Enhanced Health Promotion and Disease Prevention”, which focuses on interventions such as disease prevention, health promotion, and health protection of people living in Qatar.^15^ In addition, Qatar's National Health Strategy for 2018–2022 focuses on seven key priority areas, a crucial priority of which is “healthy and safe employees”.^16^ The MoPH has launched various healthy lifestyle programs, including a national nutrition and physical activity action plan and a wellness program at the workplace, in order to promote the health and well-being of employees.^16^

Hamad Medical Corporation (HMC) is a non-profit public healthcare provider that manages the majority of the community's healthcare needs through its healthcare facilities spread around the country.^17^ During the pandemic, HMC played a crucial role in supporting Qatar's national response to COVID-19 through the deployment of human resources, careful planning, and the implementation of innovative frameworks to address patient care challenges.^18^ The employee's perception and practice are crucial to the success of these programs. Healthcare employees play a significant role in promoting health in the community. Their lifestyle choices not only affect them personally, but also those around them. Hospital-based healthcare employees could practice health behaviors themselves. They could serve as role models and influence their patients’ and families’ attitudes toward health promotion. There is no research on hospital-based employees’ health-promoting lifestyle behaviors in Qatar. To exemplify these desirable qualities, healthcare professionals must thoroughly understand and practice health-promoting lifestyle practices. In this context, this study examined health promotion behaviors and their association with demographic characteristics among healthcare employees working at HMC, the largest secondary and tertiary healthcare provider in the State of Qatar. A comparison of health-promoting lifestyle practices between at-risk (based on the WHO risk category of NCDs) and non-risk groups of employees was conducted.

## METHODS

### Study design and settings

This was a cross-sectional study comparing the health-promoting lifestyle behaviors between at-risk and non-risk groups of employees across 14 HMC facilities, including all categories of employees.

### Participants

The organization consisted of more than 28,000 employees,^19^ and the employees working during October–November 2023 made up the study population. It included employees from various categories, including nurses, physicians, pharmacists, allied healthcare providers, accountants, educators, clerical, support, and administrative staff. The study included both males and females above 18 years of age and excluded those who had difficulty reading and understanding English. In this study, the participants were classified into “at-risk groups” according to the WHO risk category of NCDs, including those with a body mass index (BMI) >30 kg/m^2^, a smoking habit, or a pre-existing NCD, including high blood pressure, hyperglycemia, hyperlipidemia, stroke, ischemic heart disease, chronic obstructive pulmonary disease, and lung cancer.^1^

### Sample size

No previous study was conducted in the Middle East, and we assumed that 50% of the employees had good health-promoting behaviors. Based on a 95% confidence interval and 5% precision, a sample of 384 subjects was required. When considering cluster sampling, a design effect of 1.2 was applied and an additional 5% was included for the possibility of incomplete surveys, resulting in a minimum required sample size of 484.

### Recruitment

Data were collected from the participants through an online survey. Eligible subjects were invited to participate in the study through the hospital intranet and official email invitations. The link to the survey was sent to the participants along with the research information sheet. Participation in the study was voluntary, and the identity and confidentiality of the subjects were maintained throughout the study. The survey was open for two months and two reminders were sent two weeks apart to increase participation. The Microsoft form was used to collect the information from the participants. The completed surveys were included in the final analysis.

### Instrument for data collection

The English-language survey was accompanied by a research information sheet that explained the purpose of the study and ensured the confidentiality and privacy of the data. The Adolescent Health Promotion Short Form (AHP-SF) tool developed by Chen et al.^20^ was used after obtaining permission from the authors. The adolescent health promotion scale had 21 items with six subdomains, including nutrition (three items), social support (four items), health responsibility (four items), life appreciation (four items), exercise (three items), and stress management (three items). The AHP-SF showed excellent internal consistency with a Cronbach's alpha of 0.905. The AHP-SF uses a five-point Likert scale response format to obtain the information from the participants, where the response “never” was scored as 0 and “always” was scored as 4. The total scores of the AHP-SF ranged from 0 to 84. The higher value indicates better health-promoting behaviors of the participants. The demographic part of the survey included information on age, gender, educational level, nationality, occupation, BMI, smoking status, and pre-existing lifestyle diseases (NCDs).

### Data analysis

Descriptive statistics were used to summarize and determine the sample characteristics and data distribution. The mean and standard deviation (SD) were used for normally distributed data and results. The health-promoting lifestyle behaviors were calculated to summarize all the questions for different domains. Categorical data were summarized using frequencies and proportions. Associations between two or more quantitative data variables were assessed using the t-test and ANOVA, respectively. The participants were categorized into “at-risk group” and “non-risk group” and a t-test was used to assess the association. All *p* values < 0.05 were considered statistically significant. All statistical analyses were performed using the statistical package STATA version 18.

## RESULTS

A total of 526 participants completed the online survey across 14 HMC facilities. The age of the participants ranged from 22 to 69 years, with a mean age of 41 years, and the majority of them were female (64.07%). Most of the participants belonged to the age group 31–40 years (40.11%) and 41–50 years (39.54%). Approximately 43% of the participants were South Asian followed by Southeast Asian (16.15%) and Middle East Asian (14.63%). The vast majority (90.49%) of the participants had a graduate and postgraduate degree. The participants with an ideal BMI was 30.13%, and overweight and obese participants were 42.99% and 26.68%, respectively. Of the survey participants, 72.81% and 27.19% were clinical and non-clinical staff, respectively. Of the respondents, 87.64% reported that they were non-smokers and approximately 70% indicated no comorbidities. Diabetes and hypertension were the two most common comorbidities accounting for 18.06% and 9.31%, respectively (Table 1).

Table 2 shows the total score of the participants in different subdomains of the AHP-SF scale. The total score of the AHP-SF scale was 60.01/84 ± 12.32. The highest score was found in the life appreciation subscale (12.68/16 ± 2.84), followed by social support (12.03/16 ± 2.78), while the exercise subdomain (7.05/12 ± 2.93) scored the lowest. Table 3 shows the comparison of AHP-SF scores between the at-risk and non-risk groups. The total score of the AHP-SF was similar between the two groups (non-risk group: 60.12/84 ± 12.28 (95% CI 58.60091–61.65407) and at-risk group: 59.90/84 ± 12.38 (95% CI 58.43571–61.3752)). A similar pattern was observed in other subdomains, in which the exercise subscale showed the lowest score of 7.19/12 ± 3.03 in the non-risk group and 6.92/12 ± 2.84 in the at-risk group. The life appreciation subscale had the highest score in both groups (non-risk group: 12.42/16 ± 2.98 and at-risk group: 12.91/16 ± 2.69) and had statistical significance in the at-risk group (12.91/16 ± 2.69, *p* = 0.04) compared with the non-risk group (12.42/16 ± 2.98). The other five subdomains of the AHP-SF scale (nutrition, social support, health responsibility, exercise, and stress management) had no statistical significance between the non-risk and at-risk groups.

Table 4 shows the association of the demographic and clinical characteristics of the participants with total AHP-SF scores. The AHP-SF score was significantly different among the ethnic categories, with Americans having the highest AHP-SF score (63.93 ± 10.67, *p* = 0.03) and the lowest among Southeast Asians (57.70 ± 12.35). Non-smokers (60.47 ± 12.14, *p* = 0.04) had a higher AHP-SF score than ex-smokers (54.45 ± 14.56). Other demographic variables such as gender, education, occupation, age, risk category, and BMI had no statistical significance with the total AHP-SF score. Table 5 shows the total score of the participants in the 21 items of the AHP-SF scale. The highest score was obtained for items 5 and 6 in the social support subscale (3.44 ± 0.74 and 3.44 ± 1.03, respectively), whereas items 16 and 17 in the exercise subdomain had the lowest score (2.18 ± 1.20 and 2.17 ± 1.23, respectively).

## DISCUSSION

This study examined the overall practice of health-promoting lifestyle behaviors of healthcare employees in 14 hospitals in the largest public healthcare organization in Qatar. Generally, the overall health-promoting practices of the participants were at a moderate level. This is consistent with previous international studies.^21–24^ The life appreciation and social support subscales scored higher compared with stress management, nutrition, and exercises, which scored relatively low.

The present study found no significant differences between employees in the at-risk and non-risk groups in terms of health-promoting lifestyle behaviors based on the AHP-SF tool. Similarly, no statistically significant difference was found in the nutrition, social support, health responsibility, and exercise subscale scores between the non-risk and at-risk groups (Table 3). These findings indicate that participants in both groups may have similar socioeconomic status, educational level, and organizational factors that could influence their health behaviors and lifestyle choices. Moreover, workplace wellness programs launched in the organization promote health-promoting behaviors across the workforce, regardless of risk status. The influence of these factors may minimize the real differences between the at-risk and non-risk groups.

The present study showed the highest mean score in the life appreciation subscale (Table 2). Working in healthcare settings and meeting their responsibilities help them achieve self-actualization.^25^ These findings are consistent with a previous study involving nursing students who scored highest in the life appreciation subscale.^26^ These findings may reflect the participants’ self-satisfaction, accepting challenging experiences in their lives and acquiring ample opportunities for their professional and personal development.

Another important domain was social support (Table 2), which was the second highest score in the present study. The social support domain includes items such as maintaining contact with relatives, discussing concerns with others, and making an effort to build friendships. This high score in the social support domain could be due to healthy interpersonal relationships at workplace and in society. This finding is consistent with previous studies by Farokhzadian et al.^21^ and Alzahrani et al.^27^ among university staff and medical students. Previous studies among healthcare professionals found that social support is a mediator in reducing burnout and improving health in general^28^ People with greater peer support experienced less burnout compared to others.^29^

One of the important findings in this study was that most of the participants had higher scores in the health responsibility dimension (Table 2). This indicates that they were aware of the dangers of fatty and oily foods with high cholesterol content, and the importance of including fruits and vegetables in their diet (Table 5). This could be due to their constant exposure to the healthcare team, being employed in the healthcare sector, or the nature of the occupation of the participants. In addition, most of the participants were middle-aged who generally considered themselves more competent in adopting healthy behaviors. However, the participants reported a low tendency toward reporting clinical symptoms to the physician in the health responsibility domain. The possible explanation for this low tendency could be attributed to their practices, personal choices, or cultural beliefs. These results are consistent with the findings of previous studies in the Middle East, where only a small proportion of participants among medical university staff and students at health colleges and non-health colleges reported unusual signs or symptoms to their healthcare team or physician.^21,30^

In general, the results of this study indicate that the participants’ physical activity was low (Table 2). Due to the heavy workload and shift work, employees often lack physical activity. These findings are consistent with previous studies conducted among healthcare professionals,^31^ in which medical and nursing students reported low levels of physical activity.^21,22,32,33^ Mwakitalu^34^ reported that regular exercise helps reduce body weight, decrease the risk of heart disease, and maintain normal blood sugar levels. In addition, there is strong evidence that regular physical activity is important for brain health and cognition, as well as for reducing anxiety and depression and relieving stress.^35^ Overall, exercise improves the quality of life by preventing disease and enhancing well-being throughout life.^34^ Physically inactive healthcare professionals are more prone to overweight compared with others.^31^ Evidence shows that physically active healthcare professionals are more likely to advise and motivate their patients^36^ and incentives are the key facilitator in promoting physical activity.^37^ Therefore, organizations should take necessary actions to educate their employees more and develop the habit of doing simple exercise regularly in their daily lives. Moreover, physical activity, stress management, and health responsibility are important dimensions of health in preventing the development of chronic diseases.^22^ In the exercise subdomain, the lowest score was related to “Exercise rigorously 30 minutes at least 3 times per week” and “Warm up before rigorous exercise”, indicating that for most participants exercise is not a priority in their daily lives (Table 5).

Another important point in this study was stress management, which had a moderate score (Table 2). These findings are consistent with another study,^21^ which indicated that the stress levels of the participants in the present study confirmed that their stress-coping strategies could help manage the stress in their daily life and at workplace. An extensive review conducted by Kivimäki and Kawachi confirmed that workplace stress has a direct impact on increasing the risk of coronary heart disease, type 2 diabetes, and stroke.^38^ However, many strategies can be implemented in daily life to reduce and manage stress, such as meditation, yoga, and relaxation techniques. The study participants’ agreement to “make an effort to determine the source of their stress” confirms their ability to identify stress-producing factors. Employees’ wellness programs that focus on addressing the psychological needs of employees can play a vital role in stress management.

The participants of the present study had moderate scores in the nutritional domain (Table 2). However, a study of nursing students conducted in Iran found that participants had the highest mean scores in the nutrition domain.^21^ These findings indicate that participants have good awareness and knowledge of the nutritional domain. However, Mak et al.^32^ reported that nursing students followed unhealthy or unsafe methods to lose weight. The results of the present study indicate that nutritional education could be incorporated into employees’ wellness programs, which will be particularly beneficial for those with non-medical backgrounds. The participants in our study reported moderate consumption of vegetables and fruits (Table 5). These findings are in contrast with the previous study from the Middle East, where participants reported low intake of fruits and vegetables in their daily diet.^30^ The findings from a previous study show that healthcare workers who follow a shift work pattern are likely to consume more processed meat and higher amounts of animal origin fat, compared to regular daytime healthcare workers.^39^

Many factors can influence participants’ health promotion behaviors, such as education, knowledge, perceived barriers, self-efficacy, social support and situational influences,^40^ age,^21^ economic status,^24,27^ and professional category.^33^ The present study showed that the gender, educational level, occupation, age, and risk categories of the participants had no association with health-promoting behaviors (Table 4).

## STRENGTHS AND LIMITATIONS

To our knowledge, this was the first study to address the health-promoting lifestyle behaviors of healthcare employees in Qatar. The study sample consisted of all categories of employees, including non-clinical and clinical staff, working at HMC, the largest public healthcare organization in the State of Qatar. Therefore, there is a high possibility that the results presented could reflect the real picture and views of healthcare workers in Qatar. In addition, representatives from all 14 facilities participated in the study. However, the study has some limitations. The study used self-administered online surveys with convenience sampling methods, which may limit the possibilities of generalization. Another limitation is the potential for “self-selection bias”. Because the participation in the study was voluntary, it is possible that individuals who had a personal interest in well-being or were more health-conscious were more likely to participate. This could have led to an over-representation of certain attitudes or behaviors related to health and well-being, which could impact the overall findings. The cross-sectional design of the study did not explain the causes and changes in participants’ health lifestyles over time, which was another limitation.

## CONCLUSION AND RECOMMENDATIONS

The results of the present study indicate that healthcare employees working in HMC are leading a moderately healthy life, with the majority of them having higher scores in social support and life appreciation. However, the areas of nutrition, stress management, and exercise need to be improved. Developing goal-oriented health programs to encourage health responsibility among employees and regularly evaluating the effectiveness of these programs are crucial to maintaining a healthy workforce. Healthcare organizations are ideal settings to implement comprehensive workplace wellness programs and awareness campaigns that can motivate employees to take greater responsibility for their own health and influence the wider community to adopt health-promoting lifestyle behaviors. These include a healthy diet, regular exercise, stress management, preventive healthcare practices such as vaccinations and regular health screening to identify risk factors. Additionally, initiatives such as fitness challenges, nutrition workshops, and mental health support services among employees can boost their overall health-promoting behaviors. The use of digital health technologies such as mobile apps, telemedicine, or online health inquiry platforms can facilitate quick and easy access to health information and track the health progress of employees in the organization to provide personalized health support. Healthcare organizations should emphasize the importance of early detection and preventive healthcare strategies to promote the well-being of employees, including vaccinations to prevent diseases and regular health check-ups to identify potential risk factors.

### Ethical approval

Ethical approval was obtained from the IRB of HMC (protocol number MRC-01-23-508).

### Conflict of interest

The authors have no conflict of interest to declare.

### Authors’ contribution

**SR** and **NH**: literature search, survey, data collection, data analysis, and manuscript writing. **RS**: data analysis and manuscript writing. **BK**: data collection, literature search, and manuscript review. All the authors have read and approved the final manuscript.

### Acknowledgment

The authors would like to thank the participants of this study and the administration of all hospitals for their support.

## Figures and Tables

**Table 1.** Demographic and clinical characteristics of the participants.

	*n* = 526

Characteristics	Frequency (%)

Gender	

Female	337 (64.07)

Male	189 (35.93)

Age (years)	

< 30	29 (5.51)

31–40	211 (40.11)

41–50	208 (39.54)

>51	77 (14.63)

Missing	1 (0.19)

Region of origin	

Africa	35 (6.65)

America	76 (14.44)

Middle East	77 (14.63)

South Asia	227 (43.16)

Southeast Asia	85 (16.15)

Western	21 (4)

Undisclosed	5 (0.96)

Education	

Postgraduation	202 (38.4)

Graduation	274 (52.09)

Higher secondary	42 (7.98)

Secondary level and below	8 (1.52)

Occupation	

Clinical	383 (72.81)

Non-clinical	143 (27.19)

Risk category	

No-risk group	251 (47.71)

At-risk group	275 (52.28)

Body mass index (kg/m^2^)	

< 18.5	1 (0.19)

18.5–25	157 (30.13)

25–30	224 (42.99)

>30	139 (26.68)

Missing	5 (0.96)

Smoking history	

Ex-smoker	24 (4.56)

Current smoker	41 (7.79)

Non-smoker	461 (87.64)

Comorbidities	

No comorbidities	367 (69.77)

Cardiovascular diseases	13 (2.47)

Stroke	2 (0.38)

Cancer	6 (1.14)

Chronic respiratory diseases	18 (3.42)

Diabetes	49 (9.31)

Hypertension	95 (18.06)

Hyperlipidemia	27 (5.13)


**Table 2.** Total score of the participants on various subscales of the Adolescent Health Promotion Short Form AHP-SF).

Variable	Number of items	Range of score	Total score ± SD

Nutrition	3	0–12	8.31 ± 2.44

Social support	4	0–16	12.03 ± 2.78

Health responsibilities	4	0–16	11.49 ± 3.24

Life appreciation	4	0–16	12.68 ± 2.84

Exercise	3	0–12	7.05 ± 2.93

Stress management	3	0–12	8.43 ± 2.29

Total AHP-SF score	21	0–84	60.01 ± 12.32


**Table 3.** Total score of the at-risk and non-risk groups of the participants on various subscales of the Adolescent Health Promotion Short Form AHP-SF).

Variable	Non-risk group*	Total score ± SD	95% CI	At-risk group**	Total score ± SD	95% CI	*p*

Nutrition	251	8.35 ± 2.46	8.044559–8.656636	275	8.28 ± 2.42	7.995523–8.57175	0.75

Social support	251	12.01 ± 2.84	11.6624–12.36947	275	12.04 ± 2.74	11.71817–12.3691	0.09

Health responsibilities	251	11.72 ± 3.09	11.34041–12.10979	275	11.28 ± 3.37	10.88704–11.6875	0.12

Life appreciation	251	12.42 ± 2.98	12.05557–12.79702	275	12.91 ± 2.69	12.59635–13.23638	0.04

Exercise	251	7.19 ± 3.03	6.814434–7.568036	275	6.92 ± 2.84	6.589318–7.265228	0.30

Stress management	251	8.41 ± 2.27	8.135823–8.700831	275	8.44 ± 2.32	8.171183–8.723363	0.88

Total AHP-SF score	251	60.12 ± 12.28	58.60091–61.65407	275	59.90 ± 12.38	58.43571–61.3752	0.83


*Non-risk group: those with a body mass index (BMI) < 30 kg/m^2^, non-smokers, or those who do not have any pre-existing non-communicable diseases (NCDs).

**At-risk group: those with a BMI >30 kg/m^2^, smokers, or those who have any pre-existing NCDs.

**Table 4.** Association of participants’ demographic and clinical characteristics with the total Adolescent Health Promotion Short Form (AHP-SF).

Demographic	Characteristics	*N*	Total AHP-SF score (SD)	*p*

Gender	Female	337	60.10 ± 12.11	

	Male	189	59.84 ± 12.71	0.81

Education	Postgraduation	202	60.27 ± 11.01	

	Graduation	274	59.62 ± 13.16	

	Higher secondary	42	59.23 ± 12.51	0.09

	Secondary level and below	8	70.62 ± 9.36	

Occupation	Non-clinical	143	58.37 ± 13.03	

	Clinical	383	60.62 ± 12.00	0.07

Region of origin	South Asia	227	60.29 ± 13.19	

	Western	21	57.80 ± 9.85	

	America	76	63.93 ± 10.67	0.03

	Middle East	77	57.98 ± 11.28	

	Southeast Asia	85	57.70 ± 12.35	

	Africa	35	61.22 ± 10.38	

	Undisclosed	5	58.80 ± 19.71	

Age (years)	< 30	29	57.31 ± 12.13	

	31–40	211	58.74 ± 12.80	

	41–50	208	61.06 ± 12.63	0.09

	>51	77	61.59 ± 9.68	

Risk category	Non-risk group*	251	60.12 ± 12.28	0.84

	At-risk group**	275	59.90 ± 12.38	

Smoking history	Non-smoker	461	60.47 ± 12.14	

	Current smoker	41	58.07 ± 12.26	0.04

	Ex-smoker	24	54.45 ± 14.56	

Body mass index	< 25	160	60.08 ± 13.53	

(BMI, kg/m^2^)	25–30	222	60.29 ± 11.14	0.97

	>30	139	60.00 ± 12.50	


*Non-risk group: those with a BMI < 30 kg/m^2^, non-smokers, or those who do not have any pre-existing non-communicable diseases (NCDs).

**At-risk group: those with a BMI >30 kg/m^2^, smokers, or those who have any pre-existing NCDs.

**Table 5.** Adolescent Health Promotion Short Form score of the participants in various items.

	**Item**	**Response**	**Mean (SD)**

1.	I choose foods without too much oil.	526	2.80 (0.93)

2.	I include dietary fiber (e.g. fruits or vegetables).	526	2.91 (1.05)

3.	Each meal includes five food groups (e.g. bread, meat, milk, fruit, vegetable).	526	2.59 (2.59)

4.	I care about other people.	526	2.63 (1.04)

5.	I enjoy keeping in touch with relatives.	526	3.44 (0.74)

6.	I talk about my concerns with others.	526	3.44 (1.03)

7.	I make an effort to have good friendships.	526	3.25 (0. 86)

8.	I read food labels when I shop.	526	2.92 (1.03)

9.	I watch my weight.	526	3.09 (0.94)

10.	I discuss my health concerns with a doctor or nurse.	526	2.60 (1.10)

11.	I observe my body at least monthly.	526	2.88 (1.03)

12.	I usually think positively.	526	3.18 (0.85)

13.	I make an attempt to correct my defects.	526	3.18 (0.83)

14.	I make an effort to know what is important for me.	526	3.21 (0.82)

15.	I make an effort to feel interesting and challenged every day.	526	3.09 (0.86)

16.	I exercise rigorously 30 minutes at least three times per week.	526	2.18 (1.20)

17.	I warm up before rigorous exercise.	526	2.17 (1.23)

18.	I make an effort to stand or sit up straight.	526	2.69 (0.97)

19.	I make an effort to determine the source of my stress.	526	2.75 (0.93)

20.	I make schedules and set priorities.	526	2.77 (0.96)

21.	I try not to lose control when things happen that are unfair.	526	2.89 (0.89)


A five-point Likert scale was used, where “0” indicates “never” and “4” indicates “always”.
